# Oat Protein Isolate Mitigates High-Fat Diet-Induced Obesity in Rats Through Gut Microbiota, Glucose, and Lipid Control

**DOI:** 10.3390/foods14122047

**Published:** 2025-06-10

**Authors:** Xi Chen, Xue Han, Mianhong Chen, Jihua Li, Wei Zhou, Ruyi Li

**Affiliations:** 1 School of Chemistry and Chemical Engineering, Harbin Institute of Technology, Harbin 150001, China; chenxi1525@163.com; 2 Key Laboratory of Tropical Crop Products Processing of Ministry of Agriculture and Rural Affairs, Agricultural Products Processing Research Institute, Chinese Academy of Tropical Agricultural Sciences, Zhanjiang 524001, China; mianhong_chen@163.com (M.C.); foodpaper@126.com (J.L.); weizhou111@foxmail.com (W.Z.)

**Keywords:** oat protein isolate, obesity, gut microbiota, inflammation, dyslipidemia

## Abstract

The growing prevalence of obesity poses a significant challenge to public health. This research explored the impact of oat protein isolate (OPI) on mitigating obesity induced by a high-fat diet (HFD) in rats. The results indicate that OPI, administered at a dose of 100 mg/kg, reduced the gain of body weight and fat deposits, ameliorated glucose metabolism, promoted antioxidant capacity, and alleviated inflammation. Results from the 16S rRNA sequencing of fecal samples reveal that OPI mitigated the gut microbiota disorder induced by an HFD, significantly raising the proportion of beneficial genera, such as *Ruminococcus*, *Blautia*, *Allobaculum*, *Romboutsia*, and *Dubosiella*. Furthermore, OPI promoted the growth of beneficial bacteria, elevated the production of short-chain fatty acid (SCFAs), and improved glucose and lipid metabolism and reduced inflammation, collectively contributing to its anti-obesity effects. These findings confirm OPI’s efficacy in reducing obesity induced by an HFD in rats. Future clinical trials are needed to further validate the efficacy of OPI as a functional food.

## 1. Introduction

Obesity remains a widespread global health crisis [1], often attributed to excessive caloric intake and influenced by genetic, environment, and lifestyle factors [2]. Obese people are further susceptible to chronic diseases, such as diabetes, hypertension, and hyperlipidemia [3]. The incidence of obesity exhibits a rising trend based on global statistics [4]. Given the risks associated with obesity, developing effective strategies for its prevention and treatment has become increasingly urgent. Nevertheless, most anti-obesity drugs face regulatory approval challenges due to their side effects [5]. In contrast, dietary therapies, particularly those involving natural products, are rapidly gaining increased attention for managing obesity due to their minimal adverse effects and promising anti-obesity potential [6].

Numerous studies increasingly demonstrate a strong connection between obesity and gut microbiota dysbiosis [7]. Obesity is normally connected with decreased diversity in the gut microbiota [8]. This decrease in diversity is particularly evident in obesity induced by an HFD, which features an increase in *Firmicutes* and a decrease in *Bacteroidetes* [9]. Additionally, a similar phenomenon was also observed in humans, with increased *Firmicutes* and reduced *Bacteroidetes* proportions found in the intestinal flora of obese children [10]. Modulation of the gut microbiota by dietary therapies is considered an effective strategy to prevent and manage obesity [11]. Polysaccharides derived from *Artemisia sphaerocephala* Krasch seeds have been shown to mitigate obesity in mice by modulating the gut microbiota [12]. Similarly, wheat β-glucan has been demonstrated to reduce obesity and hyperlipidemia in mice through regulating the intestinal flora [13]. Lupin protein hydrolysate, rich in bioactive peptides, has been associated with gut microbiota changes that correlate with the mitigation of obesity [14]. A long-term HFD could induce dysbiosis of the gut microbiota, but supplementation with polyphenols have been shown to improve the gut microbiota composition and alleviate obesity-related symptoms in rats [15]. Therefore, maintaining a balanced gut microbiota is essential for controlling obesity and reducing the increased central appetite.

Oats are highly nutritious and rank as the fifth most consumed crop worldwide [16]. They positively impact cholesterol levels, glycemic control, and gut microbiota health [17]. With the rising demand for diverse protein sources, plant-based proteins have garnered extensive attention. Oat protein, a nutrient-rich plant protein source, offers several advantages, including a relatively high protein content, sustainable production, versatility, and low allergenicity compared with other alternative protein sources [18]. Oat protein consists of four components: globulin, albumins, prolamins, and glutenins [18]. Compared to other cereal proteins, oat protein is more nutritious, boasting higher levels of lysine and threonine and a rich profile of all eight essential amino acids [19]. Additionally, consumer acceptability scores for oat protein surpass those of other plant-based proteins, such as pea and soy proteins [16]. Oat protein exhibits lipid-lowering and antioxidant effects, positioning it as a promising health product [16]. In addition, oat peptides have been shown to markedly alleviate symptoms such as weight loss and polyphagia while aiding in blood glucose control [20,21]. The cholesterol-lowering effect of oat protein may be partially attributed to its relatively low Lys:Arg and Met:Gly ratios [22,23]. The higher arginine content in oat protein is hypothesized to exert hypocholesterolemic effects on rats induced with an HFD via normalizing HMG-CoAR activity [24]. An in vitro study has demonstrated that aromatic and hydrophobic amino acids in oat protein peptides play a significant role in reducing cholesterol levels through significantly inhibiting the activity of HMGCR and DPP4 [25]. In addition, oat protein also may reduce cholesterol levels by promoting SCFA production [26] and significantly decreasing serum low-density lipoprotein cholesterol and liver total cholesterol levels through improving the excretion of bile acids and regulating liver CYP7A1 activity [22]. However, most studies to date have been conducted in vitro, with fewer investigations assessing and focusing on the effectiveness and functional nutritional characteristics of oat protein in vivo.

In this research, the effects of OPI on obese rats fed with an HFD were preliminarily evaluated, with a subsequent focus on OPI’s anti-obesity effects on the influence of gut microbiota. This research aims to investigate the weight-loss potential of OPI and provide a theoretical support for developing functional and nutritional foods containing oat protein.

## 2. Materials and Methods

### 2.1. Materials and Chemicals

Whole oat groats were provided by Meihe Biotechnology Co., Ltd. (Xian, China). All pure analytical-grade reagents were sourced from Solarbio Science & Technology Co., Ltd. (Beijing, China).

### 2.2. Preparation of OPI

OPI was prepared based on a previous report [27]. The ultrapure water was added into defatted oat flour at a volume-to-mass of 10:1 and sufficiently mixed. The pH of acquired suspension was adjusted to 10.0, and centrifuged (4500× *g*, 30 min, 4 °C). The supernatant was adjusted to pH 5.5, followed by a second centrifugation step. The collected precipitate was dissolved and adjusted to pH 7.0, and subsequently freeze-dried. The OPI content was measured to be 90% based on a Dumas method with a nitrogen-to-protein conversion factor of 5.83 [28].

### 2.3. Animal Experimental Design

Ten-week-old male Sprague-Dawley rats, supplied by Suzhou Sibeifu Biotechnology Co., Ltd. (Suzhou, China), were housed in well-maintained animal facility under optimal hygienic conditions (12 h light/12 h dark cycle, 20 ± 1 °C). The protocol for all animal experiments was approved by the Committee of Animal Ethics of Hunter BioInsight Technology, Inc. (Hangzhou, China) and the approval number was IACUC/HTYJ-10479-01.

After one week of adaptive feeding, the rats were assigned into two treatment groups at random: the control group (CK, normal diet, *n* = 7) and the HFD-fed group (HFD, *n* = 30). The compositions of the normal diet and HFD are provided in Table S1 and Table S2, respectively. After two weeks of feeding, the serum biochemical indicators of all rats were measured and compared to those of the CK group to confirm whether the obesity model was successfully established. The obese rats were then designated into three subgroups: the model group (Mod, continuing the HFD, *n* = 7), the positive control group (PC, HFD supplemented with 50 mg/kg/day simvastatin, *n* = 7), and the OPI group (OPI, HFD supplemented with 100 mg/kg/day OPI, *n* = 7). The feeding dose of OPI (100 mg/kg/day) was calculated according to the daily consumption (50 g/day) of oats recommended by the Dietary Guidelines for Chinese Residents and the extraction yield (14.20%) of the OPI. Body weight and feed intake were determined weekly during the administration period. After six weeks of treatment, the rats were anesthetized with 1 mg/kg sodium pentobarbital, and blood and tissue samples were collected.

### 2.4. Fasting Blood Glucose (FBG) and Oral Glucose Tolerance (OGTT)

After a 12 h overnight fast, the blood glucose levels were determined in all rats at week 5 using a glucose meter (Yuyue, Jiangsu, China). Subsequently, each rat was orally administered 2 g/kg of glucose. Blood samples were gathered from the tail vein at 0, 30, 60, 90, and 120 min post-administration to measure blood glucose levels. The area under the curve (AUC) for glucose levels was obtained according to a previous reported method [9].

### 2.5. Histological Examination

Epididymal white adipose tissue (eWAT) was fixed in a fatty tissue fixative solution, while colon and liver tissues were fixed in 4% neutral paraformaldehyde. The fixed tissues were encapsulation with paraffin, and the section staining was conducted using hematoxylin and eosin (H&E). The observation and scanning of stained tissues were conducted using Pannoramic MIDI Digital Scanners (3D HISTECH, Budapest, Hungary).

Additionally, liver samples underwent Oil Red O staining to assess lipid accumulation, and representative images were captured and scanned using the same Pannoramic MIDI Digital Scanners.

### 2.6. Biochemical Analysis

The serum levels of total cholesterol (TC), triglyceride (TG), high-density lipoprotein cholesterol (HDL-C), low-density lipoprotein cholesterol (LDL-C), alanine aminotransaminase (ALT), and aspartate aminotransaminase (AST) were assessed with an 8210vet automatic biochemical analyzer (URIT, Guilin, China).

Furthermore, TC, TG, HDL-C, LDL-C, ALT, and AST of liver tissue were measured using biochemical assay kits, provided by Nanjing Jiancheng Bioengineering Institute (Nanjing, China), referring to the kit’s instruction.

### 2.7. Determination of Oxidative Stress Levels and Proinflammatory Cytokines

The liver tissue (200 mg) was homogenized with normal saline (1.8 mL), and then centrifuged (4500× *g*, 15 min, 4 °C) to gather the supernatant. The levels of malondialdehyde (MDA), myeloperoxidase (MPO), superoxide dismutase (SOD), catalase (CAT), and glutathione peroxidase (GSH-Px) in the supernatant were determined using assay kit provided by Nanjing Jiancheng Bioengineering Institute (Nanjing, China). In addition, the levels of tumor necrosis factor alpha (TNF-α), interleukin-10 (IL-10), interleukin-6 (IL-6), and interleukin-1 beta (IL-1β) were determined with enzyme-linked immunosorbent assay (ELISA) kits provided by Nanjing SenBeiJia Biological Technology Co., Ltd. (Nanjing, China).

### 2.8. Immunofluorescence Assessment

Colonic tissues were rinsed with saline and fixed with 4% neutral paraformaldehyde at 4 °C for 48 h. Running water was used to wash the tissues for 40 min, then they were gradually dehydrated in ethanol, and encapsulated with paraffin. Paraffin sections were dewaxed, rehydrated, and washed three times with PBS. Sections were treated with 3% hydrogen peroxide to suppress endogenous peroxidase activity and subsequently washed three times with PBS. The samples were then permeabilized using 0.5% Triton X-100 for 20 min, the procedure of PBS washing was repeated, and the samples were sealed by BSA at room temperature for 1 h. After additional PBS washes, the samples were incubated overnight at 4 °C with primary antibody against ZO-1 (rabbit) and Claudin-1 (mouse). Then, the secondary antibody was added to the sample and incubated for 1 h in the dark. After further PBS washes, the sections were sealed with 4,6-diamidino-2-phenylindole. Fluorescence pictures were obtained at 200× magnification using a fluorescence microscope.

### 2.9. Analysis of Gut Microbiota

An E.Z.N.A.^®^ Stool DNA Kit was applied to extract the gut microbiota DNA from fecal samples. The primers (forward primer: 5′-CCTACGGGNGGCWGCAG-3′ and reverse primer: 5′-GACTACHVGGGTATCTAATCC-3′) were designed to amplify the V3−V4 regions of fecal DNA, and then the primer sequence and PCR cycle procedure were confirmed. The high-throughput sequencing was conducted on the Illumina NovaSeq platform at Shanghai Biotree Biomedical Technology Co., Ltd. (Shanghai, China). Vsearch software (v2.3.4) was used to filter the chimeric sequence, followed by obtaining the signature table and signature sequence. The diversity indicators were calculated through QIIME (v2.0) software, and Blast (v2.0) software was used for sequence alignment. Subsequently, the signature sequence annotation was conducted by a SILVA database, and additional visualizations were created by the R package (v3.5.2).

### 2.10. Determination of Short-Chain Fatty Acids (SCFAs)

SCFAs in cecal contents were determined based on a previously established methodology with some modifications [29]. In brief, 0.5 mL of saturated NaCl solution was fully mixed with 0.05 g of the sample and incubated for 30 min. The mixture was homogenized, then 0.02 mL of 10% (*v*/*v*) H_2_SO_4_ was added and oscillated for 30 s. Diethyl ether was used for extracting SCFAs, and the mixture was centrifugated (12,000× *g*, 15 min, 4 °C). The supernatant was dehydrated using anhydrous Na_2_SO_4_. The acquired upper organic phase was collected for analysis. The SCFA content was determined by gas chromatography mass spectrometry (GCMS-QP2010 Ultra, Shimadzu, Kyoto, Japan).

### 2.11. Statistical Analysis

All data are presented as mean ± standard deviation. Statistical significance was evaluated using one-way analysis of variance (ANOVA) according to Tukey’s Honestly Significant Difference (HSD) test using SPSS 22.0. A *p*-value < 0.05 was considered statistically significant.

## 3. Results

### 3.1. Effect of OPI on Body Weight, Food Intake, eWAT, and Liver Indices of HFD-Induced Rats

**Figure 1:**
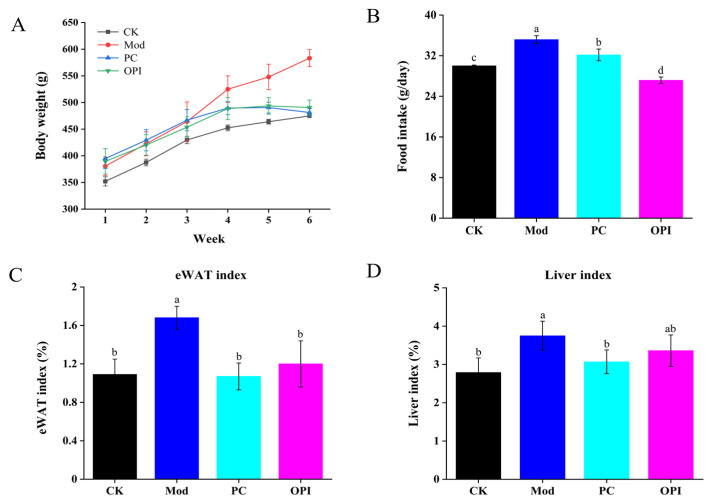
Effect of OPI on body weight (**A**), food intake (**B**), eWAT index (**C**), and liver index (**D**) in HFD-induced rats. Results are expressed as mean ± SD (n = 7), and different letters represent significant differences between different experimental groups (*p* < 0.05).

Following a 6-week intervention, body weight in the CK group was much lower than that in the Mod group (*p <* 0.001), whereas the body weights in both the PC and OPI groups were lower than that in the Mod group, with the difference of the OPI group showing a statistically remarkable difference (*p <* 0.001, Figure 1A). The food intake of the CK, PC, and OPI groups was prominently less than that of the Mod group (*p <* 0.001, Figure 1B). Moreover, OPI supplementation caused decreases in the indices of eWAT (*p <* 0.001) and liver tissue (*p <* 0.01) compared with the Mod group (Figure 1C,D).

### 3.2. Effect of OPI on Lipid Levels in Serum of HFD-Induced Rats

Serum biochemical indicators are important for diagnosing obesity-related complications. Compared to the CK group, the levels of serum TC, TG, LDL-C, HDL-C, AST, and ALT in the Mod group rats increased (Table 1), suggesting that the obesity model was established successfully. After 6 weeks of OPI administration, the levels of serum TC, TG, and HDL-C were significantly decreased compared to the Mod group (*p <* 0.05), while the LDL-C level increased. The activities of serum ALT and AST, which could reflect the metabolic function of the liver, were prominently elevated in the Mod group, suggesting inflammation and liver injury [30]. The AST/ALT ratio, a significant indicator of liver function and injury [31], was also measured. Oral OPI supplementation significantly reduced the serum ALT and AST activities, along with the AST/ALT ratio, indicating improved liver function.

**Figure 2:**
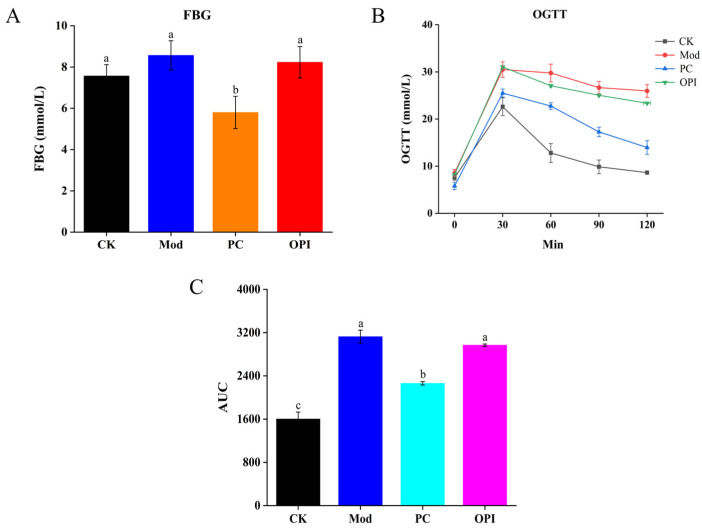
Effect of OPI on FBG (**A**), OGTT (**B**), and AUC of OGTT (**C**) in HFD-induced rats. Results are expressed as mean ± SD (n = 7), and different letters represent significant differences between different experimental groups (*p* < 0.05).

The FBG levels were much higher in the Mod group in comparison to the CK group (Figure 2A). Oppositely, the FBG levels in the PC and OPI groups were reduced by 46.55% and 3.53%, respectively, compared to the Mod group. For the OGTT, the CK group was remarkably lower than the Mod group, in addition to the PC and OPI groups (Figure 2B). Additionally, the area under the curve (AUC) in the OPI group was significantly lower in comparison to the Mod group (Figure 2C).

### 3.3. Histologic Analysis of HFD-Induced Rats Affected by OPI

**Figure 3:**
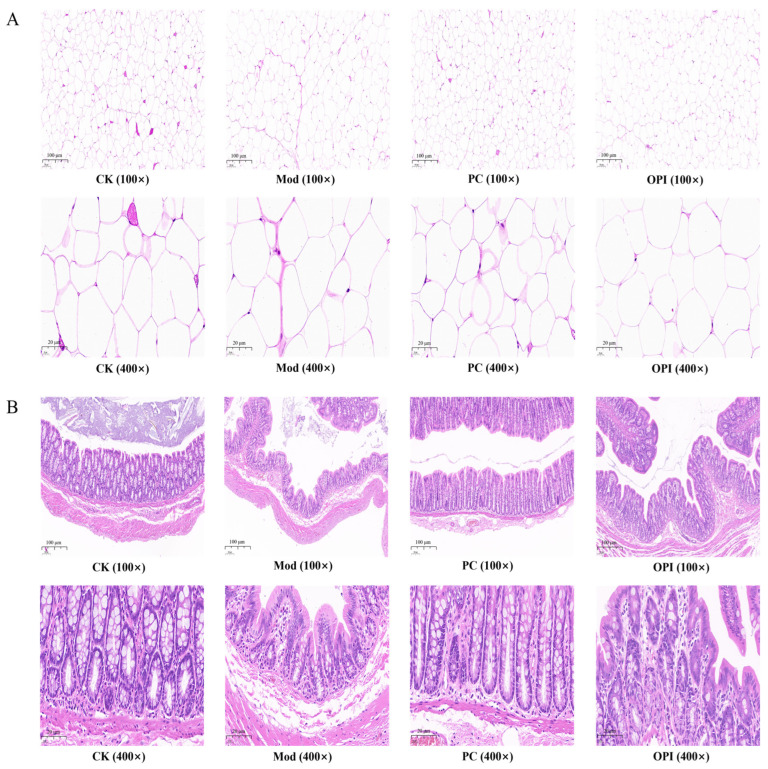
Effect of OPI on eWAT and colon histopathological features in HFD-induced rats. (**A**) Histological changes in eWAT sections were measured by H&E staining at 100× magnification and 400× magnification. (**B**) Histological changes in colon sections were measured by H&E staining at 100× magnification and 400× magnification.

The results of H&E staining for eWAT and colon tissues are shown in Figure 3. The cell morphologies of the eWAT in the CK group rats were regular, while the cell volume of the eWAT in the Mod group was obviously increased. In comparison with the Mod group, the cell volume of the eWAT in the OPI group decreased, indicating that OPI intervention alleviated the hypertrophy of eWAT cells (Figure 3A). As described in Figure 3B, the gland outline of the colon tissues in the CK group rats was clear and close, and the goblet cells were abundant and well organized. By contrast, the colon tissues of the Mod group were relatively loose, and the quantity of goblet cells was decreased. The OPI intervention obviously improved the histological structure of colon tissues in HFD-induced rats. The number of goblet cells was also significantly increased, as shown in Supplementary Figure S1A.

### 3.4. Effect of OPI on Liver Biochemical Parameters and Histopathological Features in HFD-Induced Rats

**Figure 4:**
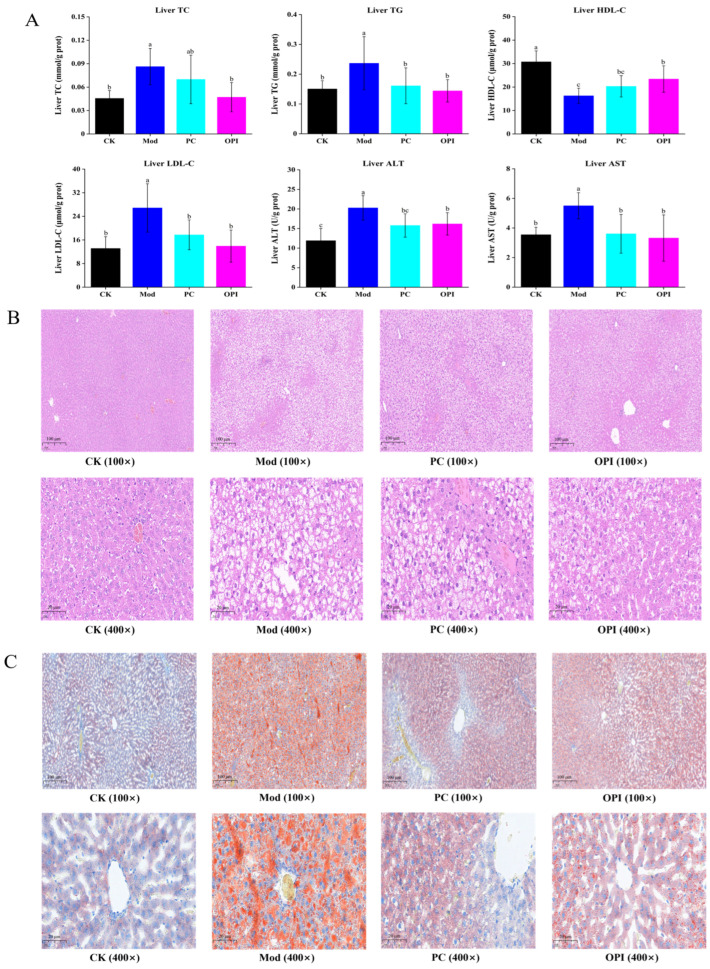
Effect of OPI on liver biochemical parameters and liver histopathological features in HFD-induced rats. (**A**) Hepatic levels of TC, TG, HDL-C, LDL-C, ALT, and AST. Results are expressed as mean ± SD (n = 7), and different letters represent significant differences between different experimental groups (*p* < 0.05). Histological changes in frozen liver sections were measured by H&E staining (**B**) and Oil Red O staining (**C**) at 100× magnification and 400× magnification.

The Mod group rats exhibited prominently higher levels of TC, TG, LDL−C, ALT, and AST compared to the CK group (*p <* 0.05, Figure 4A). These alterations were prominently suppressed by OPI intervention. The H&E staining pictures of liver tissues revealed tightly arranged hepatocytes in the CK group (Figure 4B), while in the Mod group, the hepatocytes were enlarged, disorganized, and had accumulated a large number of white vacuoles. Notably, both the PC and OPI treatments caused an obvious reduction in the number and size of lipid droplets, and markedly alleviated the excessive liver fat deposition induced by an HFD, among which the OPI group exhibited a more pronounced effect. The number of hepatocytes in the OPI group was also higher than that in the Mod group, as shown in Supplementary Figure S1B. Furthermore, the Oil Red O staining further proved that the extent of lipid accumulation in hepatocytes was significantly reduced in the OPI group compared to the Mod group (Figure 4C). Overall, these findings demonstrate that OPI effectively alleviated the HFD-induced disorders in liver lipid metabolism.

### 3.5. Effects of OPI on Liver Antioxidant Ability and Inflammatory Response of HFD-Induced Rats

**Figure 5:**
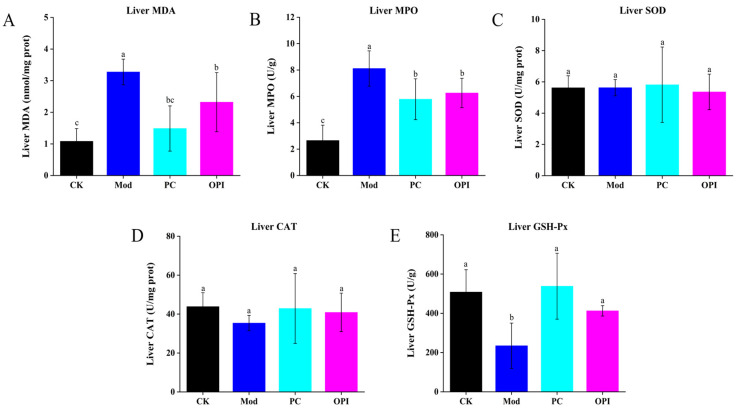
Effect of OPI on hepatic activities of MDA (**A**), MPO (**B**), SOD (**C**), CAT (**D**), and GSH-Px (**E**) in HFD-induced rats. Results are expressed as mean ± SD (n = 7), and different letters represent significant differences between different experimental groups (*p* < 0.05).

In comparison with the CK group, the Mod group exhibited prominently increased hepatic levels of MDA and MPO, alongside a marked reduction in GSH-Px activity (*p <* 0.001, Figure 5). Oral administration of OPI decreased the hepatic activities of MDA, MPO, and SOD, and increased the hepatic activity of CAT and hepatic level of GSH-Px, indicating that OPI enhances antioxidant enzyme activities and suppresses oxidative stress in HFD-induced rats.

**Figure 6:**
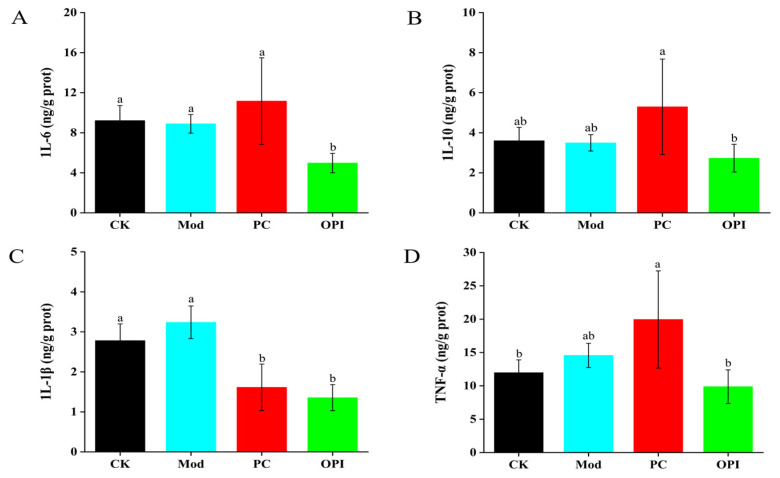
Effect of OPI on inflammatory response of IL-6 (**A**), IL-10 (**B**), IL-1β (**C**), and TNF-α (**D**) in HFD-induced rats. Results are expressed as mean ± SD (n = 7), and different letters represent significant differences between different experimental groups (*p* < 0.05).

The levels of IL-6, IL-10, IL-1β, and TNF-α in hepatocytes were measured and are summarized in Figure 6. After six weeks of the intervention, the levels of IL-6, IL-10, IL-1β, and TNF-α were significantly elevated in the Mod group. However, the intervention of OPI effectively decreased the levels of these inflammatory markers. For example, compared to the Mod group, the OPI group showed significant decreases in the levels of IL-6 (from 8.89 ± 0.92 to 4.98 ± 0.97 ng/g protein), IL-10 (from 3.50 ± 0.41 to 2.73 ± 0.69 ng/g protein), IL-1β (from 3.24 ± 0.41 to 1.35 ± 0.33 ng/g protein), and TNF-α (from 14.57 ± 1.81 to 9.87 ± 2.52 ng/g protein), and all are statistically significant (*p <* 0.05).

### 3.6. Effect of OPI on SCFA Levels in HFD-Induced Rats

**Figure 7:**
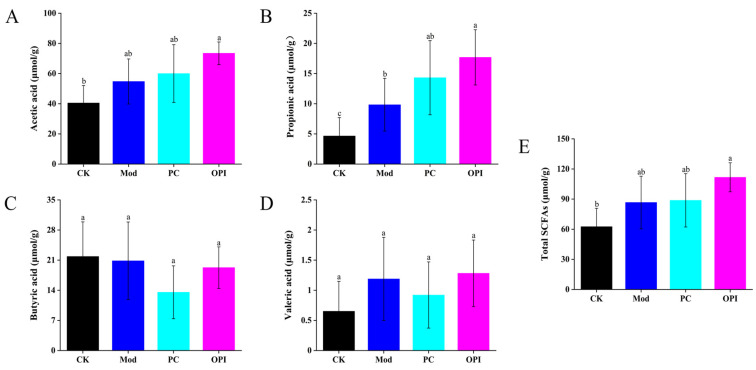
Effect of OPI on SCFAs of cecal contents in HFD-induced rats: (**A**) acetic acid, (**B**) propionic acid, (**C**) butyric acid, (**D**) valeric acid, and (**E**) total SCFA levels. Different letters represent significant differences between different experimental groups (*p* < 0.05).

SCFAs are produced from the fermentation of carbohydrates and plant-based proteins by gut microbiota in the large intestine, and are beneficial to human health [32]. Compared to the CK group, the Mod group exhibited increased levels of acetic acid, propionic acid, valeric acid, and total SCFAs in cecal contents (Figure 7). These SCFA levels were further elevated by OPI intervention, with a greater degree of enhancement than seen in the Mod group. Acetate serves as a substrate for cholesterol synthesis [33], while propionate has been shown to alleviate atherosclerosis by regulating the gut immune system and reducing vascular inflammation [34]. Furthermore, butyrate has been shown to alleviate atherosclerosis development and improve intestinal barrier function [35]. These findings suggest that OPI-mediated increases in SCFAs may contribute to its protective effects.

### 3.7. Effects of OPI on Expression of Tight-Junction Proteins in Colonic Tissues of HFD-Induced Rats

**Figure 8:**
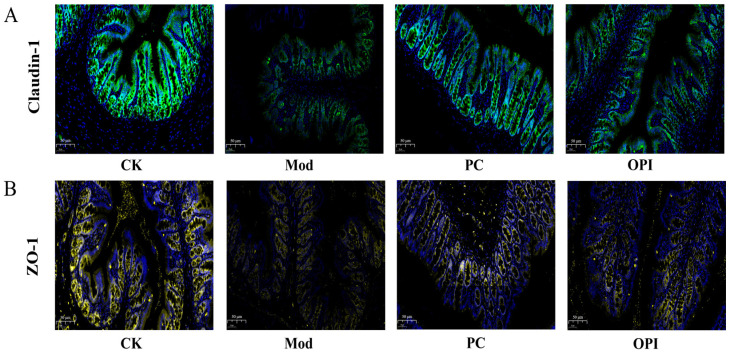
Effect of OPI on tight-junction protein expression of colonic tissues in HFD-induced rats. Immunofluorescent signals from claudin-1 (**A**) and ZO-1 (**B**) at 200× magnification.

Immunofluorescence was used for assessing the expression of tight-junction (TJ) proteins in colonic tissue samples. The fluorescent intensity of claudin-1 proteins (Figure 8A) and ZO-1 proteins (Figure 8B) in the Mod group rats were obviously weaker than those in the CK group, indicating compromised colonic barrier integrity. In contrast, the OPI intervention markedly elevated the fluorescence intensity of both claudin-1 and ZO-1 proteins, proving that OPI improves the integrity of the colonic barrier in HFD-induced rats.

### 3.8. Effects of OPI Intervention on Gut Microbiota in HFD-Induced Rats

**Figure 9:**
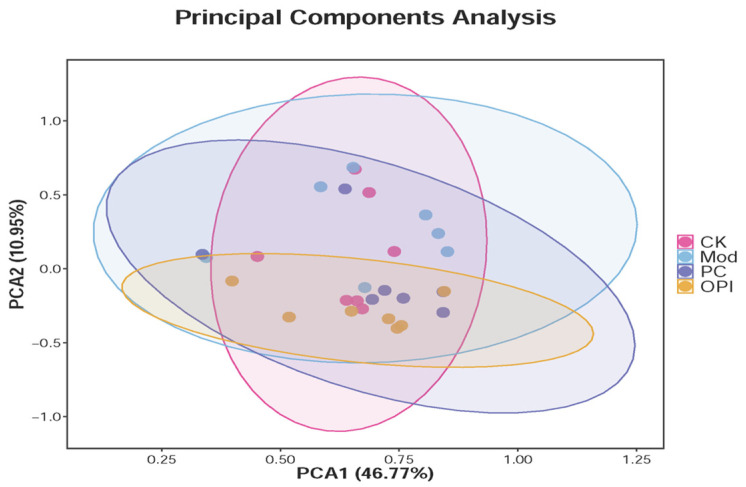
PCA score plots for CK, Mod, PC, and OPI groups on the diversity and richness of gut microbiota.

Previous research has demonstrated that the gut microbiota is essential in ameliorating glucose metabolism disorders [36,37]. To explore the impacts of OPI intervention on the gut microbiota composition in HFD-induced rats, 16S rRNA gene sequencing was conducted using fecal samples from the CK, Mod, PC, and OPI groups. PCA analysis was conducted on the relative abundance of bacterial genera. Compared to the CK group, the Mod group differed significantly, whereas the OPI group was intermediate between the CK and Mod groups (Figure 9).

**Figure 10:**
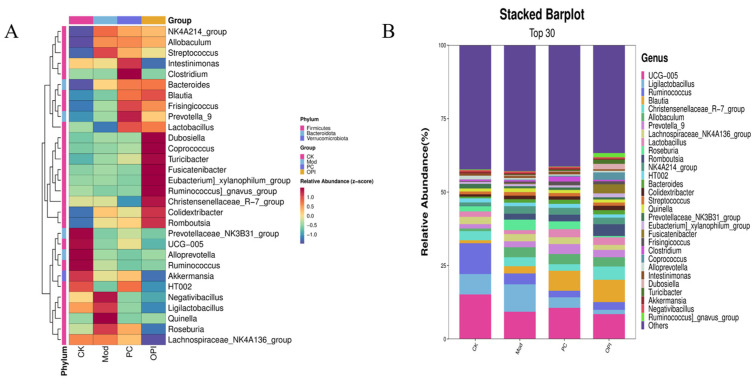
Relative abundance of gut microbiota at phylum (**A**) and genus (**B**) levels of the CK, Mod, PC, and OPI groups.

To further characterize the microbial composition, the top 30 abundant species were screened and analyzed at the phylum level (Figure 10A). In comparison with the CK group, the Mod group exhibited a reduced abundance of *Lactobacillus*, *UCG-005*, *Alloprevotella*, *Ruminococcus*, *Akkermansia*, and *HT002*, while exhibiting an increased abundance of *Quinella*, *Negativibacillus*, *Romboutsia*, *Frisingicoccus*, and *Streptococcus*. In contrast, OPI administration increased the abundance of *Colidextribacter*, *Christensenellaceae_R-7_group*, *[Eubacterium]_xylanophilum_group*, *Fusicatenibacter*, *Turicibacter*, *Coprococcus*, *Dubosiella*, *Lactobacillus*, *Blautia*, and *Bacteroides*, while reducing the abundance of *Roseburia*, *Quinella*, *Negativibacillus*, *Intestinimonas*, and *Streptococcus*. Furthermore, at the genus level, the CK group was dominated by *UCG-005*, *Ruminococcus*, *Ligilactobacillus*, *Christensenellaceae_R-7_group*, and *Lachnospiraceae_NK4A136_group*, while the Mod group was characterized by *Ligilactobacillus*, *Ruminococcus*, *Roseburia*, and *Allobaculum*. In the OPI group, *Blautia*, *Christensenellaceae_R-7_group*, *Romboutsia*, and *Allobaculum* were predominant (Figure 10B). These outcomes demonstrate that OPI treatment regulates the composition of the gut microbiota, potentially contributing to its beneficial impacts on HFD-induced metabolic dysfunction.

### 3.9. Correlation Between Intestinal Bacteria and Obesity-Related Indicators

**Figure 11:**
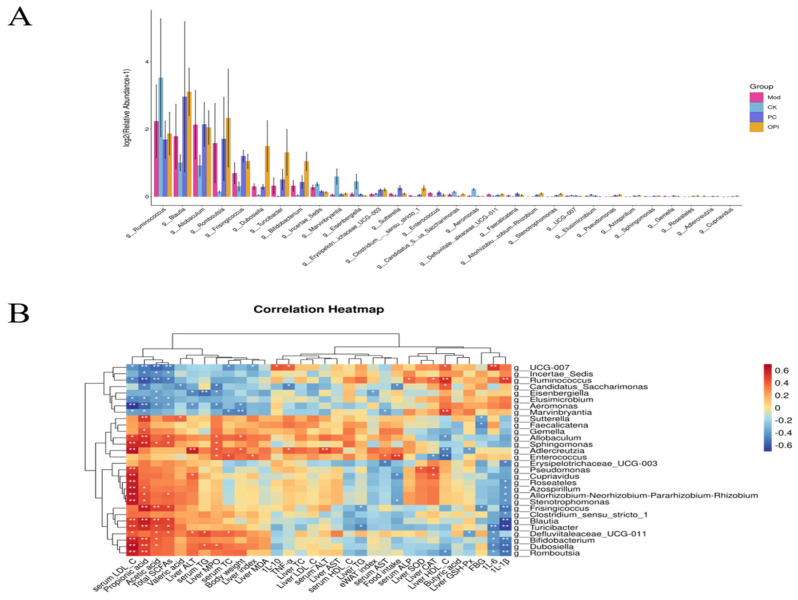
Differential microbiota at genus level of CK, Mod, PC, and OPI groups (**A**) and correlation analysis of differential microbiota with obesity related indices (**B**). The * represents *p* < 0.05 and ** represents *p* < 0.01.

As described in Figure 11A, the relative abundance of *g_Ruminococcus* in the Mod group was significantly reduced compared to the CK group, while the relative abundance of *Romboutsia* was significantly increased. The simvastatin intervention markedly elevated the relative abundance of *Blautia* compared to the Mod group while significantly decreasing the relative abundance of *g_Ruminococcus*. Interestingly, the OPI intervention significantly elevated the relative abundance of *Dubosiella* and *Turicibacter* in comparison with the Mod group, while it prominently decreased the relative abundance of *g_Ruminococcus*.

To explore the associations between obesity-related indicators and the gut microbiota, a Spearman correlation analysis was performed (Figure 11B). The results revealed that *Turicibacter*, *Blautia*, and *Frisingicoccus* exhibited positive correlations with total SCFAs while also showing negative correlations with 1L-1β levels. Furthermore, *Eisenbergiella* showed negative correlations with both liver ALT and serum TG. In contrast, *Defluviitaleaceae_UCG-011* and *Enterococcus* showed positive correlations with serum TC, whereas *Marvinbryantia* and *UCG-007* showed negative correlations with serum TC. As for body weight, *Adlercreutzia* and *Allobaculum* displayed positive associations, while *Marvinbryantia* and *Aeromonas* displayed negative associations. Noticeably, *Stenotrophomonas*, *Cupriavidus*, and *Candidatus_Saccharimonas* were negatively correlated with food intake. As for oxidative stress levels, *Enterococcus* displayed negative correlations with liver CAT, while *Defluviitaleaceae_UCG-011* and *Adlercreutzia* exhibited negative correlations with liver GSH-Px. *Frisingicoccus*, *Faecalicatena*, and *Sutterella* displayed negative correlations with FBG. Additionally, *Romboutsia*, *Dubosiella*, *Bifidobacterium*, and *Turicibacter* showed negative correlations with 1L-6 and 1L-1β levels.

## 4. Discussion

The rising global prevalence of obesity has spurred extensive research into dietary interventions, notably those utilizing natural foods. This research investigated the effects of OPI on alleviating HFD-induced obesity in rats. Following a 6-week intervention, OPI significantly decreased body weight gain, food intake, eWAT and liver tissue indices, and OGTT levels in HFD-induced rats. Nevertheless, the reduction in FBG levels in the OPI group was not significant, indicating that the effect of OPI on reducing FBG was unsatisfactory. Additionally, compared to the Mod group, the serum levels of TC, TG, and HDL-C, and the AST/ALT ratio were significantly decreased by the OPI intervention, suggesting that OPI could ameliorate disorders of glucose and blood lipids in HFD-fed rats. These results are consistent with previous studies [38,39]. HFD intake often induces the metabolism disorders of hepatic lipids. Compared with the Mod group, OPI effectively reduced hepatic lipid levels. Moreover, the results of the H&E and Oil Red O staining further confirmed that OPI markedly alleviated excessive hepatic lipid accumulation caused by an HFD, thereby improving hepatic lipid metabolism. These positive impacts might be due to the antioxidant properties of OPI. Obesity-related complications are often directly or indirectly associated with oxidative stress [40]. OPI enhanced the hepatic activity of CAT and hepatic level of GSH-Px in HFD-fed rats, while decreasing the hepatic activities of MDA and MPO. Hepatic antioxidant enzymes play a critical role in promoting the liver’s lipid metabolism, thereby preventing fat accumulation [41]. Previous studies have demonstrated that oat proteins and peptides possess prominent antioxidant capacities [42,43]. Additionally, the correlation between lipid metabolism disorders and inflammation is well-documented [44]. In the present study, OPI showed strong anti-inflammatory effects by prominently decreasing the levels of IL-6, IL-10, IL-1β, and TNF-α in the liver. These findings are consistent with reports that oat bioactive peptides can modulate immune responses by enhancing innate and adaptive immunity [16].

Growing evidence indicates that SCFA production is vital for human health, enhancing lipid and glucose metabolism while supporting immune balance [32]. Some studies also have indicated that SCFAs can help alleviate atherosclerosis [45], hypertension [46], and pancreatic and colon injury [47]. Additionally, SCFA supplementation has been reported to significantly benefit the treatment of Crohn’s disease and ulcerative colitis [48]. In this study, the OPI group exhibited higher cecal concentrations of acetic acid, propionic acid, valeric acid, and total SCFAs than the Mod group.

Extensive evidence indicates a significant connection between the gut microbiota and obesity [49]. In this study, the OPI group showed increased relative abundances of *Colidextribacter*, *[Eubacterium]_xylanophilum_group*, *Coprococcus*, *Lactobacillus*, *Blautia*, and *Bacteroides* compared to the Mod group. Increasing evidence suggests that *[Eubacterium]_xylanophilum_group* promotes SCFA production, which supports intestinal health and immune homeostasis [50]. *Lactobacillus*, widely known for its health benefits, is commonly used as a probiotic. Recent reports confirm that *Lactobacillus* can decrease the levels of LDL-C and TC in obese adults [51] and mitigate obesity in HFD-induced rats by improving lipid metabolism and regulating leptin and adiponectin levels [52]. Additionally, *Blautia* is negatively connected with the visceral fat area, which is strongly linked to cardiovascular disease [53]. *Blautia* has been shown to improve diabetes indices, regulate the Th17/Treg balance, and mitigate inflammation [54]. Similarly, *Bacteroides* is negatively correlated with obesity [55].

Overall, OPI significantly reshaped the gut microbiota by promoting the beneficial bacterial growth associated with weight management, thereby contributing to its anti-obesity effects. The close relationship between obesity and inflammation is well established [9]. In our study, HFD feeding induced inflammation in rats, whereas OPI supplementation significantly increased the relative abundances of *Turicibacter* and *Blautia*, which were positively associated with total SCFAs, while being negatively associated with 1L-1β levels. These findings suggest that OPI supplementation decreases inflammation by regulating the gut microbiota. Notably, *Allobaculum* was significantly more plentiful in the Mod group and was positively associated with body weight. Additionally, *Romboutsia*, *Dubosiella*, and *Turicibacter* correlated negatively with 1L-6 and 1L-1β levels, further confirming that OPI mitigates HFD-induced inflammation through gut microbiota regulation.

## 5. Conclusions

This study indicates that OPI reduces obesity in HFD-induced rats by decreasing body weight gain, decreasing eWAT and liver indices, ameliorating glucose and lipid disorders, elevating antioxidant capacity, and alleviating inflammation. Furthermore, OPI plays a significant role in reshaping the gut microbiota and increasing its richness and diversity, enhancing the abundance of beneficial bacteria associated with obesity alleviation. This study confirms OPI’s potential for treating obesity and associated metabolic disorders in rats induced by an HFD. Future research should explore the molecular mechanisms by which OPI ameliorates host obesity from a multi-omics perspective.

## Figures and Tables

**Table 1:** Effect of OPI on serum biochemical parameters in HFD-induced rats.

Groups	TC (mmol/L)	TG (mmol/L)	LDL-C (mmol/L)	HDL-C (mmol/L)	AST (U/L)	ALT (U/L)	AST/ALT
CK	0.64 ± 0.11 ^c^	1.19 ± 0.39 ^b^	0.33 ± 0.08 ^b^	1.27 ± 0.22 ^a^	134.57 ± 15.15 ^b^	56.00 ± 9.09 ^a^	2.50 ± 0.52 ^a^
Mod	1.68 ± 0.08 ^a^	2.30 ± 0.38 ^a^	0.95 ± 0.42 ^a^	1.46 ± 0.30 ^a^	236.43 ± 45.35 ^a^	74.43 ± 15.41 ^a^	3.30 ± 0.89 ^a^
PC	0.71 ± 0.06 ^bc^	1.34 ± 0.46 ^b^	0.74 ± 0.24 ^a^	1.41 ± 0.28 ^a^	144.86 ± 23.86 ^b^	60.29 ± 15.50 ^a^	2.50 ± 0.70 ^a^
OPI	0.79 ± 0.10 ^b^	1.98 ± 0.41 ^a^	1.11 ± 0.23 ^a^	1.30 ± 0.39 ^a^	138.86 ± 46.91 ^b^	62.14 ± 26.49 ^a^	2.33 ± 0.51 ^a^

Results are expressed as mean ± SD (n = 7), and different letters represent significant differences between different experimental groups (*p* < 0.05).

## Data Availability

The original contributions presented in this study are included in the article/Supplementary Materials. Further inquiries can be directed to the corresponding author.

## Supplementary Materials

The following supporting information can be downloaded at https://www.mdpi.com/article/10.3390/foods14122047/s1, Table S1: Composition and energy distribution of normal diet used in animal experiment; Table S2: Composition and energy distribution of high-fat diet used in animal experiment; Figure S1: Number of goblet cells in colon H&E staining at 400× magnification (A) and hepatocytes in liver H&E staining at 400× magnification (B).
