# Supplementary material for: Oat Protein Isolate Mitigates High-Fat Diet-Induced Obesity in Rats Through Gut Microbiota, Glucose, and Lipid Control
Source: Foods. 2025 Jun 10;14(12):2047. doi: 10.3390/foods14122047 (PMC12192181; doi:10.3390/foods14122047)
Supplement: Supplementary file 1 [file foods-14-02047-s001.zip › foods-3669920-supplementary.pdf]

Supplementary Table S1. Composition and energy distribution of the normal diet used in the animal experiment.

Ingredients	%	Nutritional Components	%
Casein	22	Protein	23
Corn starch	24	Fat	20
Maltodextrin	8	Carbohydrate	57
Sucrose	14		
Cellulose	6		
Cacao oil	17		
Mix mineral	6		
Mix vitamin	3		

Supplementary Table S2. Composition and energy distribution of the high-fat diet used in the animal experiment.

Ingredients	%	Nutritional Components	%
Basic diet	65	Protein	15.6
Oil	10	Fat	34.8
Sucrose	20	Carbohydrate	49.6
Cholesterol	2.5		
Sodium cholate	1		
Mix mineral	1		
Mix vitamin	0.5		

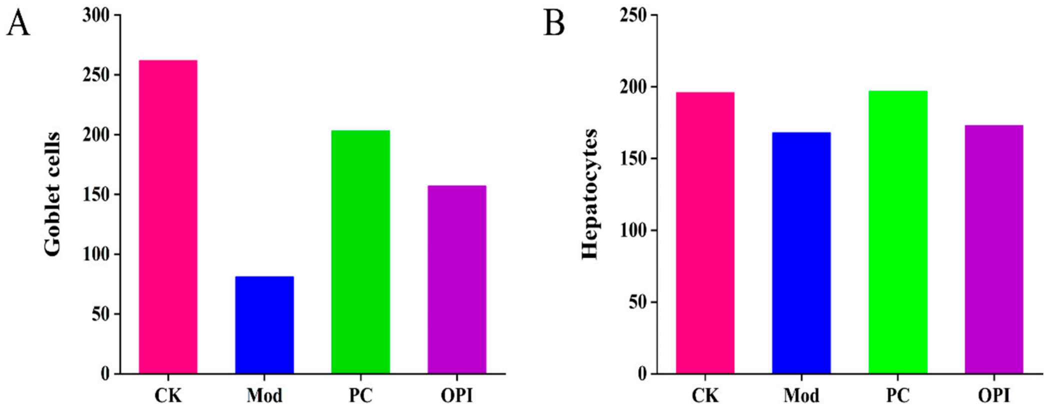

Supplementary Figure S1. The number of goblet cells in colon H&E staining at 400× magnification (A) and hepatocytes in liver H&E staining at 400× magnification (B).
